# Association of elevated levels of plasma chloride, in severity and mortality, in adult patients in the ICU

**DOI:** 10.1186/cc14440

**Published:** 2015-03-16

**Authors:** M Aguilar Arzapalo

**Affiliations:** 1 Hospital O'Horan, Mérida, Mexico

## Introduction

For a long time, many investigators have tried to demonstrate increased mortality associated with acid-base disturbances. In this study, we sought to determine the association of hyperchloremia measured at ICU admission and whether this electrolyte disturbance is associated with an increase in morbidity and mortality.

## Methods

Data were retrospectively collected for consecutive adult patients admitted to Agustin O'Horan Hospital ICU, between January 2011 and July 2014, who underwent inpatient medical treatment using electronic files.

## Results

**Figure 1:**
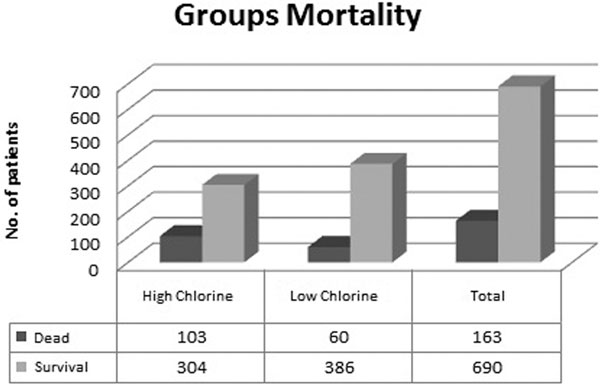
**Group mortality, high and low chlorine**.

The dataset consisted of 936 medical files and serum chloride concentration values on admission, 853 being eligible. Hyperchloremia (serum chloride >110 mmol/l) is quite common, with an incidence of 47.71%. Patients were propensity matched based on their association with death and hyperchloremia. Of the 853 patients collected, patients with hyperchloremia after admission (*n *= 446, 52.3%), patients were matched to patients who had normal serum chloride levels after admission. These two groups were well balanced with respect to all variables collected. The hyperchloremic group was at increased risk of mortality at ICU discharge, relative risk ratio = 1.81; 95% confidence interval, 1.41 to 2.51 risk increase of 25.31%. Admission hyperchloremia was associated with increased morbidity, mortality and higher scores in severity scales; this association was statistically important. See Figure 1.

## Conclusion

This retrospective cohort trial demonstrates an association between hyperchloremia and poor ICU admission outcome (death). Additional studies are required to demonstrate a causal relationship between these variables.
